# Effect of rumen-protected standardized dry grape extract supplementation on antioxidant and immune-related gene expression in leukocytes of mid-lactating Fleckvieh cows exposed to natural heat stress

**DOI:** 10.3168/jdsc.2026-1068

**Published:** 2026-05-30

**Authors:** Annalisa Amato, Carmelo Cavallo, Paul Engler, Hoa Bui, Mohammed el Amine Benarbia, Andrea Minuti, Erminio Trevisi, Anna Antonella Spina, Concetta Molè, Maria Giovanna Ciliberti, Marzia Albenzio, Luigi Liotta, Vincenzo Lopreiato

**Affiliations:** 1 Department of Veterinary Sciences, Messina University, 98168, Messina, Italy; 2 Nor-Feed, 49070 Beaucouzè, France; 3 Department of Animal Science, Food and Nutrition (DIANA), Faculty of Agricultural, Food and Environmental Sciences, Università Cattolica del Sacro Cuore, 29122 Piacenza, Italy; 4 Interdepartmental Services Centre of Veterinary for Human and Animal Health (CISVetSUA), Magna Græcia University of Catanzaro, 88100 Catanzaro, Italy; 5 Department of Agriculture, Food, Natural Resources and Engineering (DAFNE), University of Foggia, 71121 Foggia, Italy

## Abstract

Heat stress (HS) disrupts oxidative homeostasis and impairs immune function in dairy cows. In this context, plant polyphenols, when included in the diet, may modulate antioxidant defenses and immune response, including the antioxidant ability of leukocytes. This study evaluated whether supplementation with rumen-protected standardized dry grape extract (Nor-Grape BP-O; Nor-Feed) modulates antioxidant and immune-related gene expression in circulating leukocytes of mid-lactating Fleckvieh cows under natural HS. Thirty cows were randomly assigned to a control diet (CTR; n = 15) or to the same diet supplemented with 470 mg/cow per day of Nor-Grape BP-O (NG-BPO; n = 15) and monitored for 35 d during summer. Blood samples were collected at 0, 17, and 35 d for leukocyte isolation and quantitative PCR analysis. Relative gene expression was calculated using LinReg software and then transformed into ln fold change (lnFC) relative to 0 d. Data were subjected to PROC GLIMMIX procedure of SAS version 9.4 (SAS Institute). Overall, NG-BPO cows showed higher lnFC expression of glutathione peroxidase 1 (*GPX1*) and glutathione-disulfide reductase (*GSR*) than CTR cows. The lnFC superoxide dismutase 1 (*SOD1*) expression tended to be greater in NG-BPO cows compared with CTR cows, whereas superoxide dismutase 2 (*SOD2*) expression was unaffected by the supplementation. Among immune-related genes, interleukin-1β (*IL1B*) had an overall greater lnFC expression in NG-BPO cows, particularly at d 35, when a heat wave occurred, compared with CTR cows. A similar pattern was observed for lnFC interleukin-10 (*IL10*) expression, which was greater at 35 d in NG-BPO cows compared with CTR cows. In contrast, toll-like receptor 2 (*TLR2*), myeloid differentiation primary response 88 (*MYD88*), interleukin-1 receptor (*IL1R*), and glucose transporter 3 (*GLUT3*) were not affected by supplementation. The early upregulation of *GPX1* and *GSR* at 17 d suggests a transitory activation of oxidative detoxification pathway driven by polyphenols, followed by downregulation at 35 d. Concomitantly, the increased *IL10* and *IL1B* expression at 35 d in NG-BPO cows may suggest an enhanced capacity to regulate inflammatory signaling and maintain immune homeostasis, by preventing the negative effect of excessive immune activation. Overall, rumen-protected standardized dry grape extract supplementation was associated with a positive modulation of leukocyte genes involved in antioxidant defense system and regulation of inflammation, providing new molecular insight supporting previously reported improvements in physiological and immunometabolic responses during HS.

## Highlights

• Rumen-protected standardized grape extract modulates leukocyte gene expression under HS.
• Nor-Grape BP-O increased *GSR* and *GPX1* expression, enhancing antioxidant defense.
• *IL1B* and *IL10* mRNA expression in supplemented cows suggests improved immune homeostasis.

## Graphical Abstract

**Figure:**
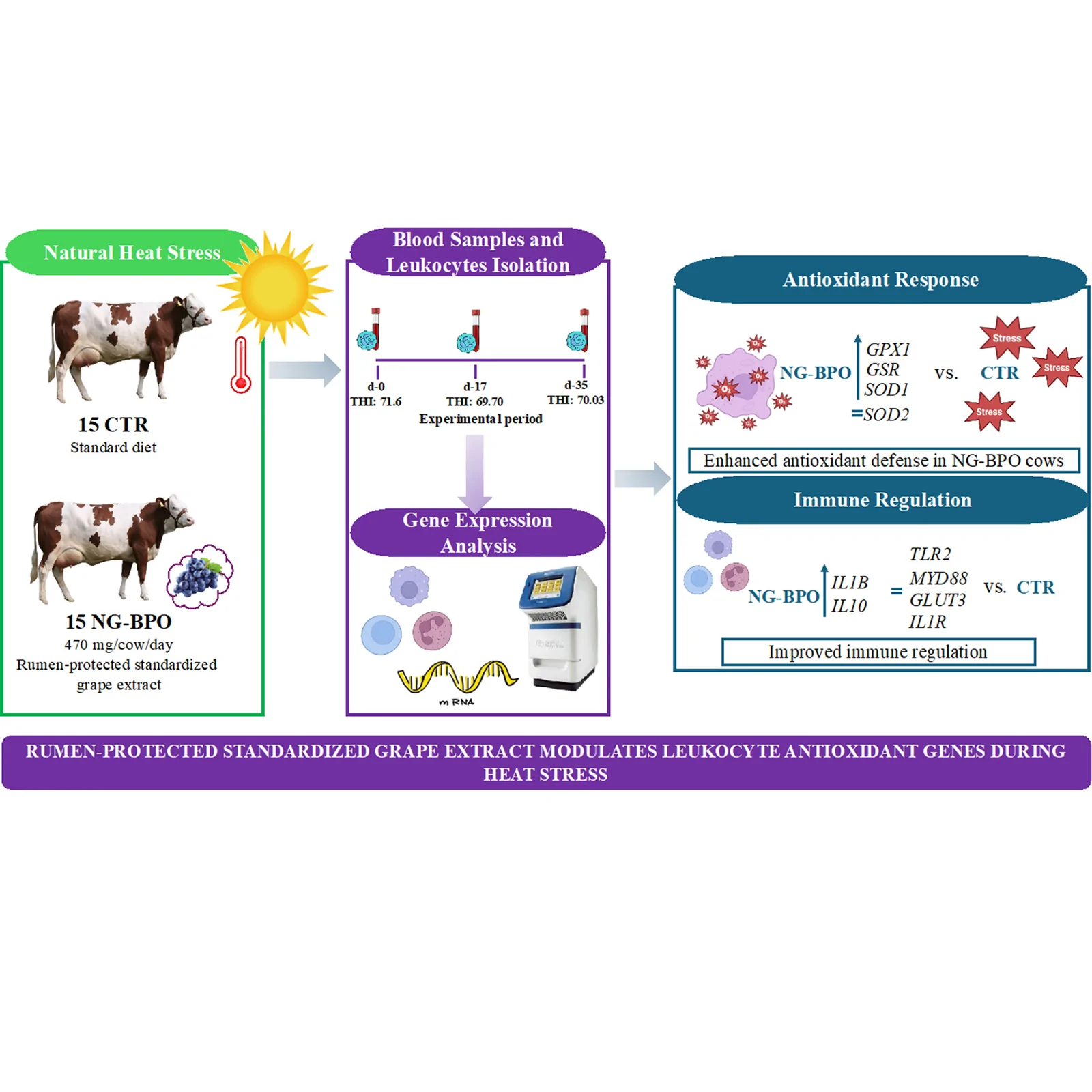
**Summary:** This study evaluated the effects of rumen-protected standardized dry grape extract supplementation on leukocyte gene expression in Fleckvieh cows under heat stress (HS). Thirty cows (15/group) were assigned to a control diet (CTR) or to a diet supplemented with 470 mg/cow per day of rumen-protected dry grape extract (NG-BPO) for 35 days. Blood samples were collected at 0, 17, and 35 days to assess antioxidant and immunerelated gene expression. Supplementation increased *GPX1* and *GSR* mRNA expression, indicating enhanced antioxidant defense, particularly at 17 days. During a heat wave at 35 days, supplemented cows showed greater *IL1B* and *IL10* mRNA expression, suggesting improved immune regulation. Overall, standardized grape extract supplementation supported leukocyte adaptation to heat stress by modulating antioxidant and inflammatory response. The graphical abstract was created by the authors using Microsoft PowerPoint and BioRender.com. THI = temperature-humidity index.

Heat stress (**HS**) is a major challenge for the dairy sector, impairing productivity, reproduction, immune response, and inflammation (Polsky and von Keyserlingk, 2017), particularly during summer months. One of the primary biological consequences of HS is the disruption of redox homeostasis (Chauhan et al., 2014), which occurs because of an imbalance between the production of reactive oxygen species (**ROS**) and the antioxidant defense mechanism (e.g., enzymatic antioxidant, such as SOD and GPX, and nonenzymatic antioxidant, including vitamins and GSH; Loor et al., 2023). Although ROS are essential for the physiological function of cells, their excessive production results in damage of macromolecules, lipids, and proteins and also in oxidative stress (Sordillo and Aitken, 2009). Several studies have shown that specific leukocyte subpopulations, such as peripheral blood mononuclear cells and polymorphonuclear leukocytes, are highly sensitive to heat challenges, reducing proliferation (Lacetera, 2019), phagocytosis, and oxidative burst under in vitro studies (Lecchi et al., 2016). These functional impairments are associated with alterations in intracellular redox balance and stress-responsive transcriptional pathways, suggesting that leukocyte gene expression profiles represent an early and sensitive marker of immune adaptation to HS, as reported by Lopreiato et al. (2020). Nutritional supplementation, such as dietary antioxidants, can mitigate oxidative stress exposition through free radicals scavenging activity, thus, reducing or preventing oxidative damage (Chauhan et al., 2014). Plant polyphenols are known to exert anti-inflammatory effects and to improve oxidative status (Gessner et al., 2017; Nair et al., 2025). Among the different plant polyphenols resources, grape supplementation may represent an effective strategy during HS to counteract oxidative stress, while supporting the immune response. In our recent study (Amato et al., 2025), the supplementation of 470 mg of rumen-protected standardized dry grape extract (282 mg of rumen-protected polyphenols/head per day) in dairy cows under HS showed better thermoregulation, reflected by lower rectal temperature and reduced heavy breathing. Moreover, immunometabolic responses improved as well, by registering higher plasma zinc, globulin (likely reflecting a greater immunoglobulin availability), myeloperoxidase, and neutrophil and monocyte phagocytic activity, lower haptoglobin, and modulating inflammatory cytokines during the peak of HS. Although several studies have evaluated the inclusion of grape pomace in dairy cow diets, evidence regarding the effects of grape-derived polyphenols on leukocyte gene expression during HS is still limited. Moreover, available studies have typically used high doses of unprotected, nonstandardized polyphenols, which are more susceptible to ruminal degradation, less available for intestinal absorption, and may consequently lead to inconsistent outcomes. Based on previous findings, the aim of this study was to evaluate changes in the expression of genes related to inflammation, immune function, and antioxidant defense in blood leucocytes of Fleckvieh cows supplemented with rumen-protected standardized dry grape extract (Nor-Grape BP-O, Nor-Feed), under naturally occurring HS.

The experimental procedures were approved by the University of Messina Animal Care Committee (protocol 06/2024 bis) and followed ARRIVE guidelines. The experimental trial was previously published in Amato et al. (2025). Briefly, during peak summer (July–August), under natural HS conditions, a total of 30 Fleckvieh (dual-purpose Simmental) primiparous and multiparous (parity 2–6) mid-lactation (170 ± 61 DIM) cows were randomly assigned according to milk yield, parity, and DIM to dietary groups fed for 5 consecutive weeks with (1) standard milking cow diet (**CTR**, n = 15) or (2) standard milking cow diet supplemented with 470 mg/d of Nor-Grape BP-O (a rumen-protected standardized dry grape extract rich in water-soluble polyphenols; Nor-Feed, France; **NG-BPO**, n = 15). The supplementation of NG-BPO was mixed with corn meal (100 g) and top-dressed individually onto TMR once a day in the morning at 0700 h and provided a daily dietary supply of 282 mg bioactive polyphenols per cow. The CTR group received, instead, an equivalent amount of corn meal as a placebo. All cows were housed in the same pen and fed ad libitum once a day as TMR with a common diet (Amato et al., 2025). All the animals of both groups were cooled all days. The temperature-humidity index (**THI**) was calculated based on the equation reported by Ouellet et al. (2021). The daily mean, maximum, and minimum THI registered throughout the trial are reported in the Figure 1. After 25 d of treatment, the THI registered was above 70 until 33 d of treatment, identifying that period of increased THI as a heat wave according to Hahn (1999), who defined a heat wave as at least 3 d with a mean THI ≥ 70. Blood samples for whole blood leukocytes isolation were collected at 0, 17, and 35 d of treatment. Blood samples were collected by jugular venipuncture into 10-mL heparinized tubes (BD Vacutainer; Becton, Dickinson and Co., Franklin Lakes, NJ) before the morning feeding and immediately placed in ice water. After collection, blood leucocytes were collected by centrifugation (800 × *g* for 5 min at 4°C) and isolated by washing samples with a red blood cell lysis buffer until a white blood cell pellet was obtained followed by the addition of 1 mL of QIAzol (Qiagen, Hilden, Germany). Samples were stored at −80°C. The total RNA was then extracted using the RNeasy Mini Kit (Qiagen, Hilden, Germany), and residual DNA was removed using the RNase-Free DNase Set (Qiagen, Hilden, Germany), following the manufacturer's protocols. After extraction, RNA was quantified using the Qubit RNA BR Assay Kit (Thermo Fisher Scientific, Waltham, MA) and RNA quality was assessed using the Bioanalyzer 2100 with RNA 6000 Nano Labchips according to the manufacturer's instructions (Agilent Technologies, Dublin, Ireland). The average of the RNA quality index for the samples was 9.1 (range: 6.5–9.6). Synthesis of cDNA was performed using a reverse transcription kit (RevertAid RT Reverse Transcription Kit; Thermo Fisher Scientific, Monza, Italy), as previously described. The resultant cDNA was diluted 1:4 (vol/vol) with DNase/RNase-free water and then stored at −80°C before quantitative PCR (**qPCR**) testing. The qPCR was performed using 8 μL of diluted cDNA combined with 12 μL of a mixture composed of 10 μL of SYBR Green master mix (Applied Biosystems, Foster City, CA), 0.8 μL each of 10 μ*M* forward and reverse primers, and 0.4 μL of DNase/RNase-free water in a MicroAmp Optical 96-Well Reaction Plate (Applied Biosystems). Each sample was run in triplicate. The qPCR procedure was performed using the ABI Step-one real-time PCR System (Applied Biosystems, Warrington, UK) using the following conditions: 2 min at 50°C, 10 min at 95°C, 40 cycles of 15 s at 95°C (denaturation), and 1 min at 60°C (annealing + extension). The analyzed genes included 3 internal control genes (β-actin [*ACTB*], glyceraldehyde-3-phosphate dehydrogenase [*GAPDH*], and tyrosine 3-monooxygenase/tryptophan 5-monooxygenase activation protein zeta [*YWHAZ*]). Genes selected for transcript analysis were those related to antioxidant response, glutathione peroxidase 1 (*GPX1*), glutathione reductase (*GSR*), superoxide dismutase 1 (*SOD1*), and superoxide dismutase 2 (*SOD2*)]; immune mediation and inflammatory cascade functions, toll-like receptor 2 (*TLR2*), myeloid differentiation primary response 88 (*MYD88*), interleukin-1β (*IL1B*), interleukin-1R (*IL1R*), and interleukin-10 (*IL10*); and glucose uptake, glucose transporter 3(*GLUT3*). The qPCR efficiency and quantification cycle values were obtained for each reaction using LinRegPCR (Version 2017.1; Amsterdam UMC, Amsterdam, the Netherlands). The final data were normalized using the geometric mean of 2 internal control genes (*ACTB* and *GAPDH*), identified as the most stable reference genes by the geNorm algorithm. Normalized gene abundance data were used to calculate fold change (**FC**) in mRNA abundance at d 17 and 35 relative to baseline (d 0). Fold change was computed as the ratio between mRNA abundance at each time point and the corresponding baseline value. The resulting FC values were log-transformed using the natural logarithm (ln) to improve normality. Statistical analyses were performed on ln-transformed FC (**lnFC**) values using repeated measures mixed models with the GLIMMIX procedure of SAS version 9.4 (SAS Institute Inc., Cary, NC). The model included treatment, time, and their interaction as fixed effects and cow as a random effect. Time (day) was included as repeated measure within cow. Least squares means were compared using the pairwise differences (PDIFF) statement. Significance was declared at *P* ≤ 0.05 and tendency at 0.05 < *P* ≤ 0.10.

**Figure 1:**
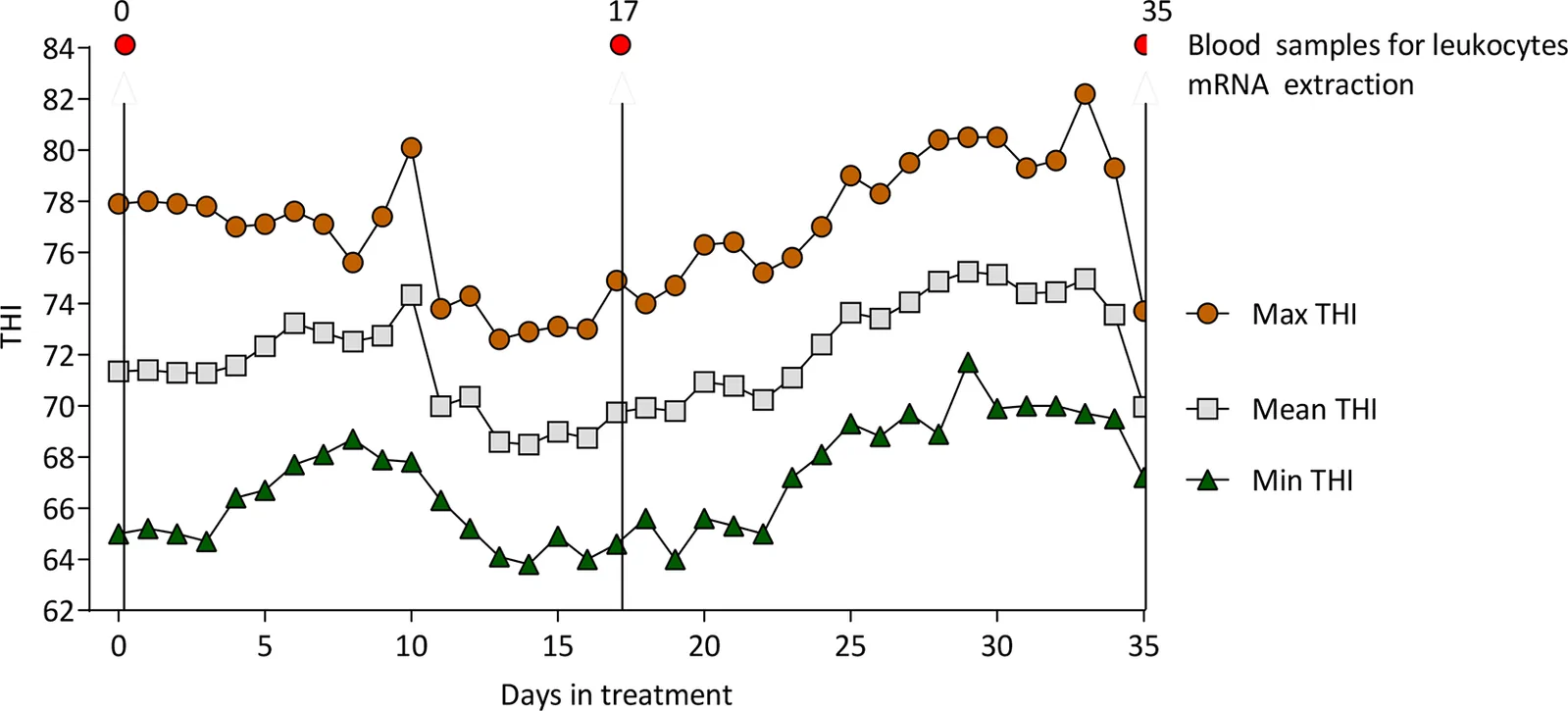
Experimental design with sampling time points and THI trends of all lactating cows enrolled in the trial under the same environmental conditions, throughout the experimental entire period (during summer). Max = maximum; Min = minimum.

The expression of genes involved in oxidative stress is reported in Figure 2. Overall, lnFC *GPX1* expression was greater in NG-BPO compared with CTR cows (treatment, **Trt**; *P* < 0.05). In particular, *GPX1* was upregulated relative to baseline at 17 d of supplementation in NG-BPO cows, showing higher lnFC expression than CTR cows (Trt × time; *P* < 0.05), whereas *GPX1* expression was downregulated relative to baseline in CTR cows. Moreover, *GPX1* mRNA expression was downregulated in both groups at 35 d of treatment (Figure 2). A similar pattern was observed for *GSR*, whose expression was overall greater in NG-BPO cows compared with CTR cows (Trt; *P* < 0.05). In addition, *GSR* was upregulated at 17 d in NG-BPO cows and showed greater expression compared with CTR cows (Trt × time; *P* < 0.05), which, in contrast, was downregulated. However, at 35 d, *GSR* expression was downregulated in both groups (Figure 2).

**Figure 2:**
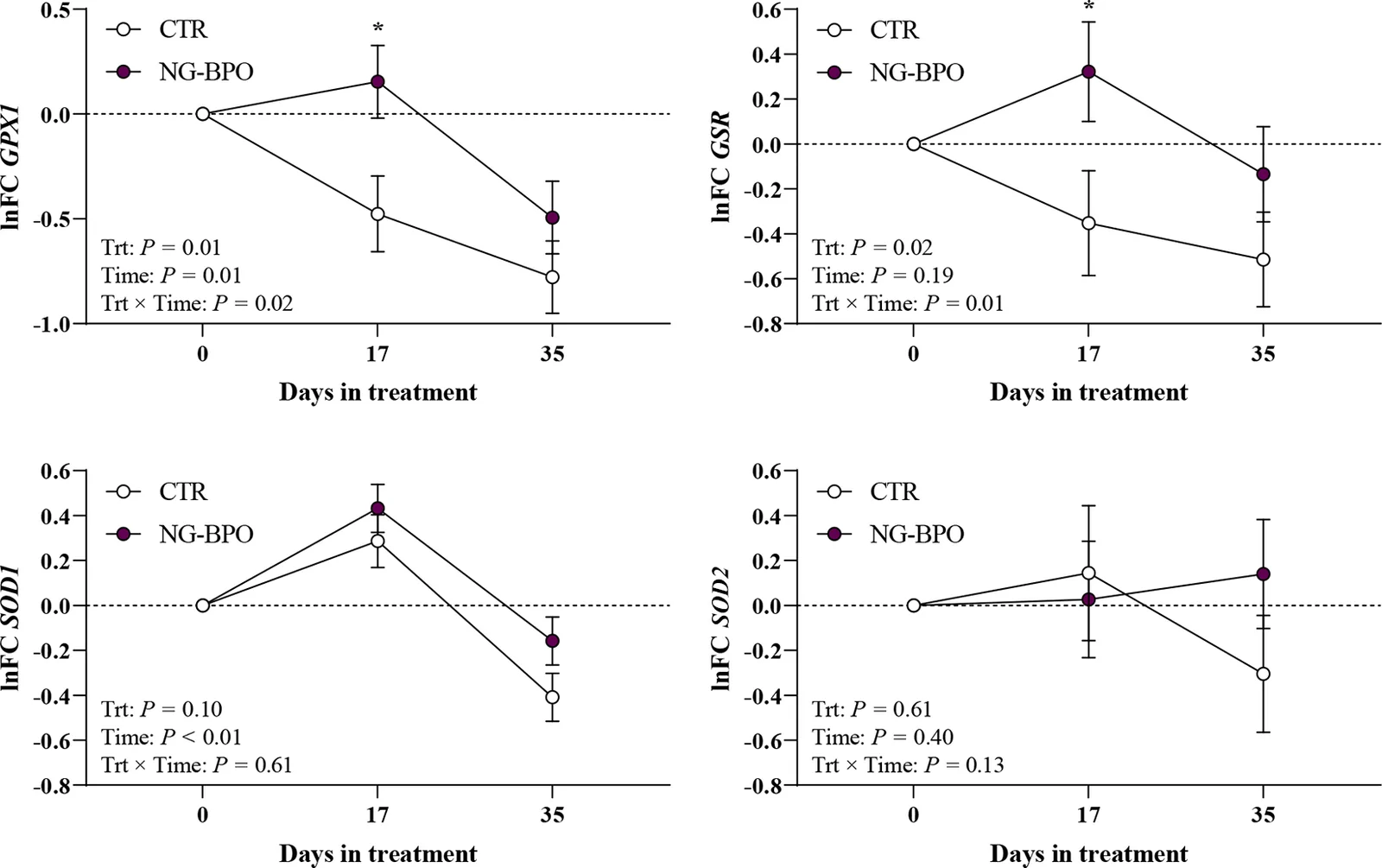
Changes in leukocyte mRNA expression of antioxidant-related genes (lnFC) *GPX1*, *GSR*, *SOD1*, and *SOD2* of mid-lactating Fleckvieh cows fed either a standard diet (CTR) or a diet supplemented with rumen-protected dry grape extract at 470 mg/head per day (NG-BPO) for 35 d during a natural HS exposure in summer. Differences between groups within each time point (*P* ≤ 0.05) are denoted with an asterisk. Error bars represent SE.

Regarding lnFC *SOD1* expression, it tended to be overall greater in NG-BPO cows (Trt; *P* = 0.10). In particular, it was upregulated at 17 d relative to baseline in both groups, followed by downregulation at 35 d.

In contrast, lnFC *SOD2* expression was not affected by NG-BPO supplementation (*P* > 0.05). Glutathione peroxidase 1 (GPX1), is a selenoprotein associated with antioxidant function (Smith et al., 1997). Indeed, its role is related to the conversion of ROS into less reactive metabolites by using GSH, thus protecting tissue against oxidative damage and preventing peroxidation of membrane lipids (Papp et al., 2007). In this study, the upregulation of *GPX1* in the NG-BPO group may be associated with the ability of polyphenols in stimulating the endogenous antioxidant system, which is important to promote during an early stage of stress condition. Indeed, previous studies reported that polyphenols can upregulate the nonenzymatic (e.g., glutathione, GSH) and the enzymatic (e.g., GPX, SOD, CAT) antioxidant defense system (Rather and Bhagat, 2020; Guo et al., 2023), thereby supporting immune cell function and resilience to oxidative challenges. The elimination of ROS is also achieved by glutathione reductase (GSR) and superoxide dismutase (SOD). Glutathione reductase plays a key role in maintaining intracellular redox balance by converting the oxidized glutathione (GSSG) to its reduced form (GSH) using NADPH (Yan et al., 2012), thereby sustaining cellular peroxide detoxification and protecting cells from oxidative injury (Reed, 1969; Cohen et al., 1987). Superoxide dismutase represents the first line of antioxidant defense, having a key role in catalyzing the dismutation of superoxide into hydrogen peroxide, which must then be neutralized by second-line enzymes such as catalase and GPX (Rinaldi et al., 2007). Glutathione reductase supports this system as a third line of defense, by recycling GSH and maintaining redox capacity. Under the moderate heat load (17 d), NG-BPO cows showed greater *GPX1* and *GSR* expression, consistent with an enhanced ability to activate endogenous antioxidant defenses during HS. In contrast, the generalized reduction in gene expression at 35 d was not unexpected, given the severe heat wave registered in that period. Notably, the less pronounced alterations observed in NG-BPO cows indicate a partial protective effect of rumen-protected polyphenols, suggesting improved resilience despite the inability to fully counteract extreme HS. In this study 2 isoforms of SOD were evaluated: SOD1, which resides in cytoplasm and SOD2, which resides in mitochondria; all of them require catalytic metal (Cu or Zn) for their activation (Fukai and Ushio-Fukai, 2011). Although no significant differences between groups were observed for *SOD2* expression, the observed tendency for greater *SOD1* expression in NG-BPO cows may be linked to increased zinc availability. Indeed, zinc is an essential cofactor for SOD1 activity and structural stability (Fukai and Ushio-Fukai, 2011), and its circulating concentration is typically reduced during inflammatory conditions because of hepatic sequestration during the acute-phase response (Rink and Kirchner, 2000). Therefore, the greater Zn availability previously observed in NG-BPO cows by Amato et al. (2025) may have contributed to supporting SOD1 expression and cytosolic antioxidant defense. Moreover, the combination between *SOD1* expression together with the increased expression of *GPX1* and *GSR* suggests a coordinated stimulation of multiple endogenous antioxidant defense lines in supplemented cows. At least in nonruminants, the transcription regulator nuclear factor erythroid 2 like 2 (NFE2L2) is considered the key regulator of cellular redox balance and can induce the expression of antioxidant and detoxification enzymes and downstream phase-II enzymes such as SOD and GPX (Sies and Jones, 2020). Recently, the direct effect of plant polyphenols on the NFE2L2-oxidative stress response of dairy cows was established, as also reviewed by Guo et al. (2023). Indeed, Daddam et al. (2023) reported that supplementation with plant polyphenol extracts increased the abundance of proteins associated with the Nrf2-mediated oxidative stress response pathway, including heme oxygenase 1 (HMOX1) and microsomal glutathione S-transferase 2 (MGST2), in adipose tissue of heat-stressed dairy cows. In particular, MGST2 is involved in glutathione-dependent detoxification processes, supporting the activation of pathways related to glutathione (GSH) redox homeostasis (Oakley, 2005). Hence, the greater FC observed in *GPX1* and *GSR*, together with the tendency toward increased *SOD1* expression, could potentially reflect modulation of this transcriptional pathway and the endogenous antioxidant defense response.

The lnFC *IL1B* expression was overall greater in NG-BPO cows compared with CTR cows (Trt; *P* < 0.05), with a significant Trt × time interaction (*P* < 0.05). In detail, as shown in Figure 3, *IL1B* expression was upregulated at 17 d relative to baseline in both groups, whereas at 35 d of supplementation it was downregulated in both groups, with a less pronounced reduction in NG-BPO cows compared with CTR cows (Trt × time; *P* < 0.05). However, lnFC *IL10* expression was downregulated in both groups and tended to be overall higher in NG-BPO cows compared with CTR cows (Trt; *P* = 0.10), with a significant Trt × time interaction, shown by higher expression in NG-BPO cows at 35 d of supplementation (*P* < 0.05; Figure 3).

**Figure 3:**
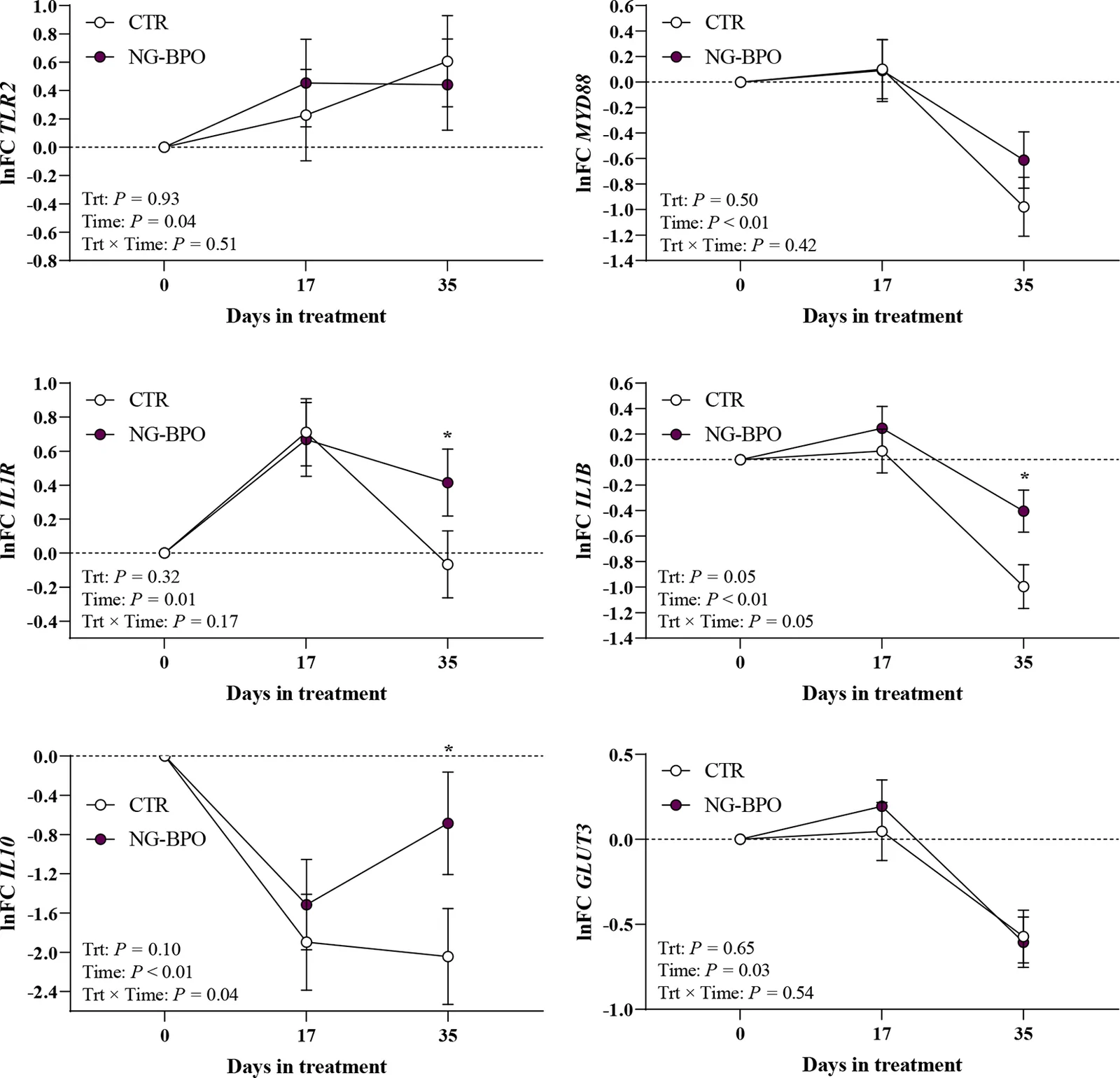
Changes in leukocyte gene expression of immune- and inflammation-related genes (lnFC) *TLR2*, *MYD88*, *IL1R*, *IL1B*, *IL10*, and energy-related gene *GLUT3* of mid-lactating Fleckvieh cows fed either a standard diet (CTR) or a diet supplemented with rumen-protected dry grape extract at 470 mg/head per day (NG-BPO) for 35 d during a natural HS exposure in summer. Differences between groups within each time point (*P* ≤ 0.05) are denoted with an asterisk. Error bars represent SE.

Interleukin-1β (IL-1β) is a key proinflammatory cytokine involved in the activation of innate immune responses, contributing to leukocyte recruitment and functional activation of neutrophils and monocytes, as well as to the enhancement of phagocytic activity (Peker and Musal, 2022). Previous in vitro studies conducted under HS conditions, have reported a reduction in IL-1β production and secretion by isolated polymorphonuclear leukocytes, a response associated with the downregulation of immune- and oxidative stress-related pathways and a consequent impairment of immune competence (Lopreiato et al., 2020). In this context, the higher *IL1B* expression observed in NG-BPO cows may indicate the preservation of a controlled and physiologically relevant inflammatory status, rather than an excessive or pathological inflammatory activation, which is required for the proper regulation of both innate and adaptive immune functions. This finding is consistent with the previous evidence reporting increased IL-1β levels in plasma of NG-BPO cows exposed to HS (Amato et al., 2025), suggesting that polyphenols may contribute to modulation of IL-1β–mediated immune responses. In contrast, IL-10 is a key anti-inflammatory cytokine involved in limiting the degree of inflammatory response, thereby preventing excessive tissue damage (Bazzoni et al., 2010; Zhou et al., 2015). Because IL-10 regulates the production of both pro- and anti-inflammatory cytokine, the greater *IL10* expression observed at 35 d of supplementation in NG-BPO cows may reflect an improved ability to modulate inflammatory signaling and maintain immune homeostasis, especially during the heat wave registered in the last days of the trial. Similarly, polyphenol-enriched wine lees extracts tested in an in vitro ovine model of inflammation increased IL-10 secretion while reducing IL-1β production (Ciliberti et al., 2022), thereby contributing to a more balanced pro- to anti-inflammatory cytokine ratio. Indeed, from a physiological standpoint, inflammation is a response with the aim to identify and eliminate a threat. Overall, the results of gene expression FC in this study indicate that the greater FC expression of genes associated with inflammation and immune regulation (*IL10* and *IL1B*) and antioxidant system (*GPX1, GSR*, and *SOD1*) in NG-BPO cows could be related to an enhanced ability to cope with oxidative and inflammatory challenges during HS and during a heat wave, consistent with a modulatory effect of grape polyphenol supplementation on leukocyte stress-responsive pathways. In contrast, the lack of treatment effects on upstream innate immune signaling genes (*TLR2*, *MYD88*) and on *IL1R* suggests that the level of HS exposition did not activate pathogen-recognition pathways, like in circulating leukocytes. Rather, in circulating leukocytes the transcriptional response appeared selective, mainly involving antioxidant-related genes and cytokine regulation, consistent with a modulatory—rather than stimulatory—effect on immune function (Cavaillon, 2001). Moreover, the faster kinetics of the SOD-GSH antioxidant system, mainly observed at 17 d, compared with the later cytokine modulation detected at 35 d, suggests that leukocytes may prioritize the restoration of redox homeostasis during HS adaptation rather than the prompt activation of immunomodulatory mechanisms. The rapid activation of antioxidant defense systems may represent a protective strategy to limit redox-driven activation of inflammatory cascades during HS adaptation. Therefore, the timing of adaptation to HS may partly reflect the earlier activation of antioxidant defenses relative to the subsequent modulation of cytokine responses. According to previous findings, tannin supplementation in mid-lactating dairy cows enhanced plasma antioxidant capacity, reduced oxidative stress markers, and modulated the proinflammatory cytokine profile, thereby contributing to the prevention of immune depression during the summer season (Santillo et al., 2022). Consistently, Amato et al. (2025) reported that supplementation with rumen-protected standardized dry grape extract increased monocyte and neutrophil phagocytic activity, further supporting the role of grape-derived polyphenols in promoting immune competence under HS conditions. Thus, the highlighted biological effect on gene expression related to antioxidant defense and inflammation may be associated with rumen protection, which likely reduces ruminal degradation and enhances systemic availability, thereby improving the efficiency of polyphenol supplementation. Notably, the present study provided only 282 mg of bioactive polyphenols/cow per day, whereas previous studies using unprotected grape-derived products reported substantially greater amounts, ranging from approximately 6,240 to 32,540 mg/cow per day (Gessner et al., 2015; Ianni et al., 2019).

Supplementation with rumen-protected standardized dry grape extract appears to be a useful nutritional strategy to support production, behavioral responses, and immune status of dairy cows under HS. Notably, the dose of rumen-protected polyphenols used in the present study (470 mg/d) was markedly lower than amounts reported in previous studies. Together, these results indicate that the supplementation is associated also with selective transcriptional changes in genes related to circulating leukocytes involved in oxidative stress and inflammation.
